# Supplementary material for: Molecular pathway and structural mechanism of human oncochannel TRPV6 inhibition by the phytocannabinoid tetrahydrocannabivarin
Source: Nat Commun. 2023 Aug 2;14:4630. doi: 10.1038/s41467-023-40362-2 (PMC10397291; doi:10.1038/s41467-023-40362-2)
Supplement: Supplementary file 1 — Supplementary Information [file 41467_2023_40362_MOESM1_ESM.pdf]

Supplementary Information

Molecular pathway and structural mechanism of human oncochannel TRPV6 inhibition by the phytocannabinoid tetrahydrocannabivarin

Arthur Neuberger[1], Yury A. Trofimov[2], Maria V. Yelshanskaya[1], Jeffrey Khau[1], Kirill D. Nadezhdin[1], Lena S. Khosrof[1], Nikolay A. Krylov[2], Roman G. Efremov[2], Alexander I. Sobolevsky[1,*]

[1] Department of Biochemistry and Molecular Biophysics, Columbia University, New York, NY, USA

[2] Shemyakin-Ovchinnikov Institute of Bioorganic Chemistry, Russian Academy of Sciences, Moscow, Russia

* Corresponding author. Tel: +1 2123054249; E-mail: **as4005@cumc.columbia.edu**

This PDF file includes:

Supplementary Figures 1-8

Supplementary Table 1

Supplementary References

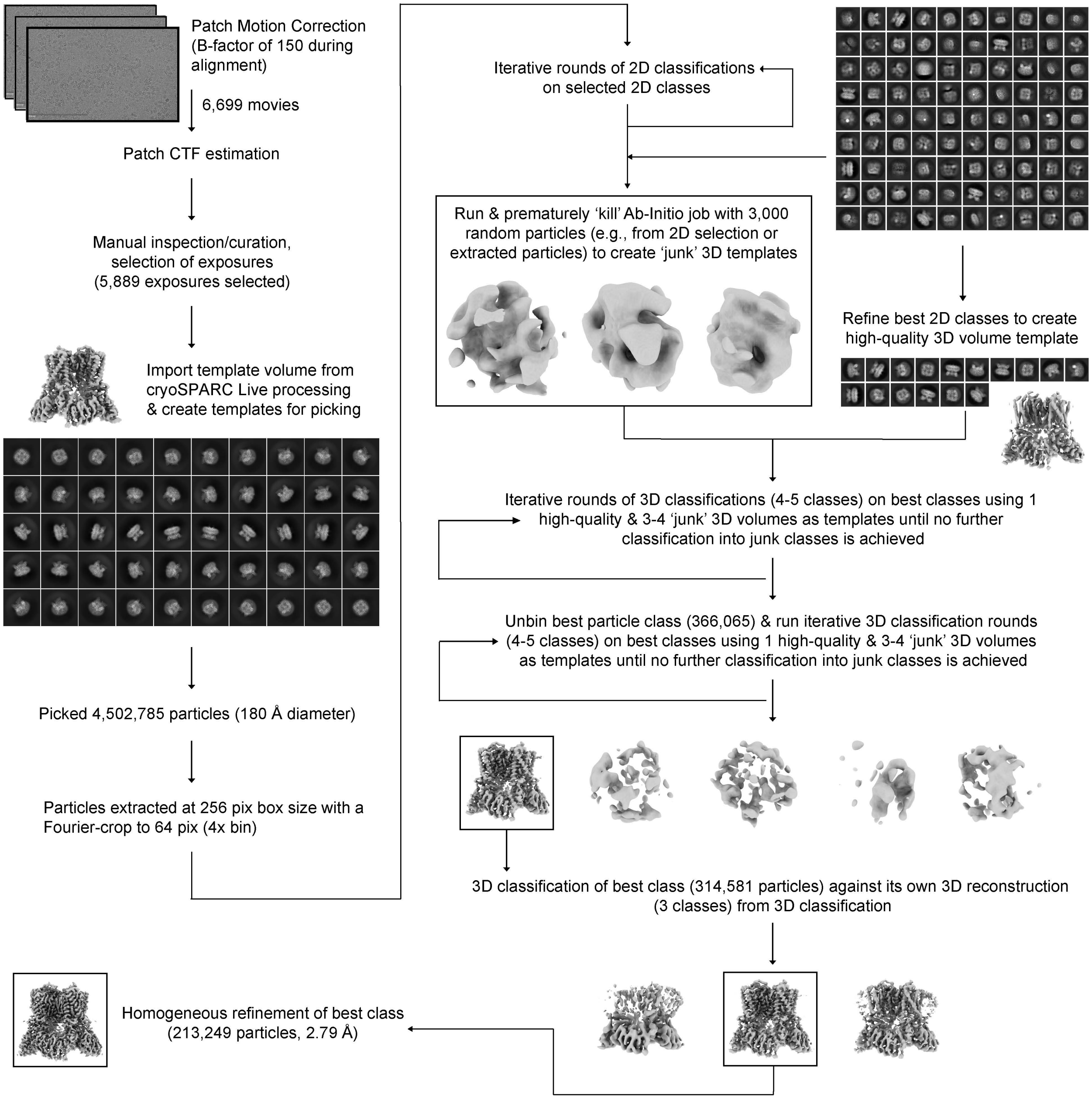

Supplementary Fig. 1 | 3D reconstruction workflow for hTRPV6$_{THCV}$. Examples of 2D class averages and 3D volumes are shown at different stages of cryo-EM data processing workflow.

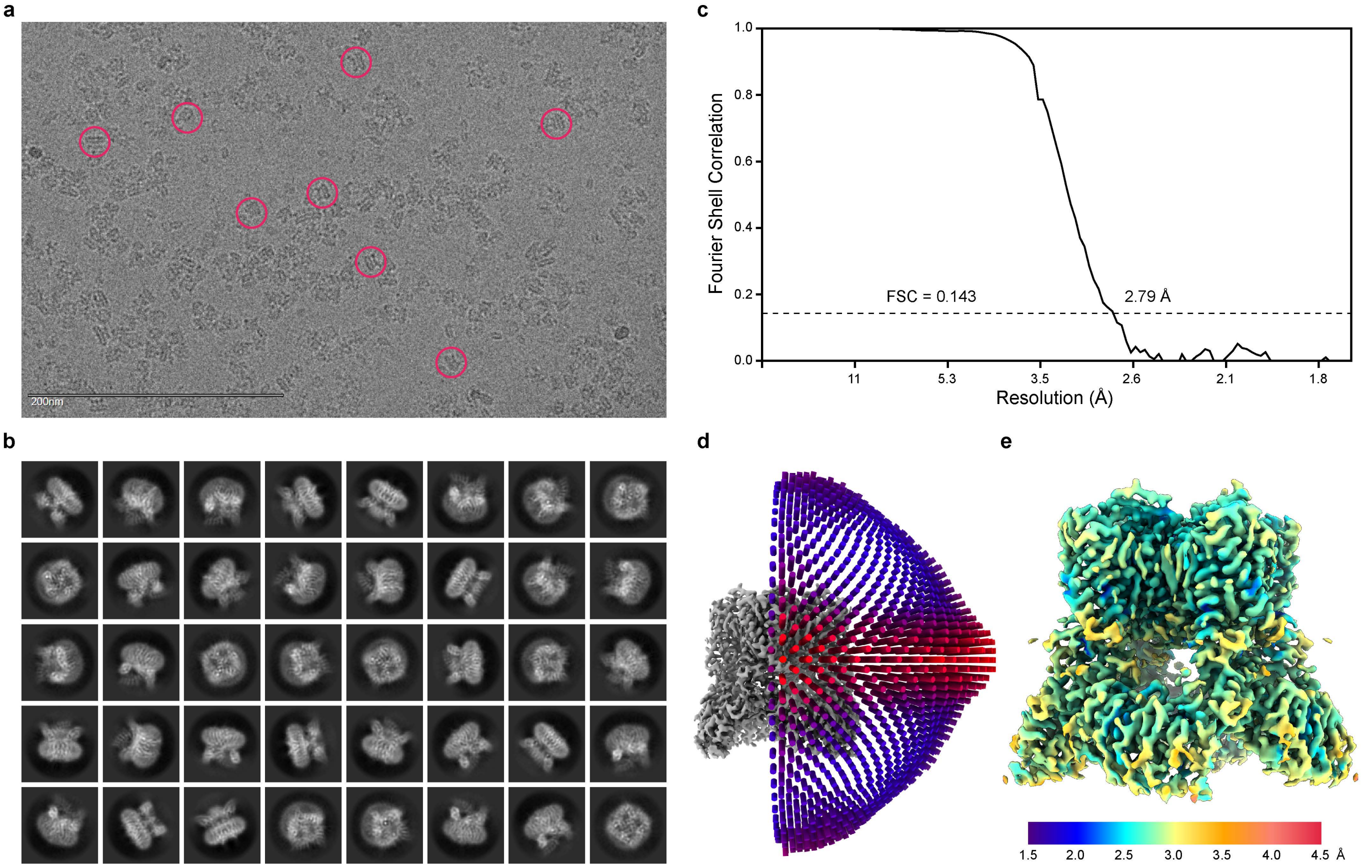

Supplementary Fig. 2 | Overview of cryo-EM data for hTRPV6$_{\mathbf{THCV}}$. a Representative micrograph with example particles circled in pink. A total number of 6,699 such micrographs were collected. **b** Representative 2D class averages. **c** FSC curve. **d** Euler angle distribution of particles contributing to the final reconstruction with larger red cylinders representing orientations comprising more particles. **e** Local resolution presented as coloring of the TRPV6$_{THCV}$ cryo-EM map.

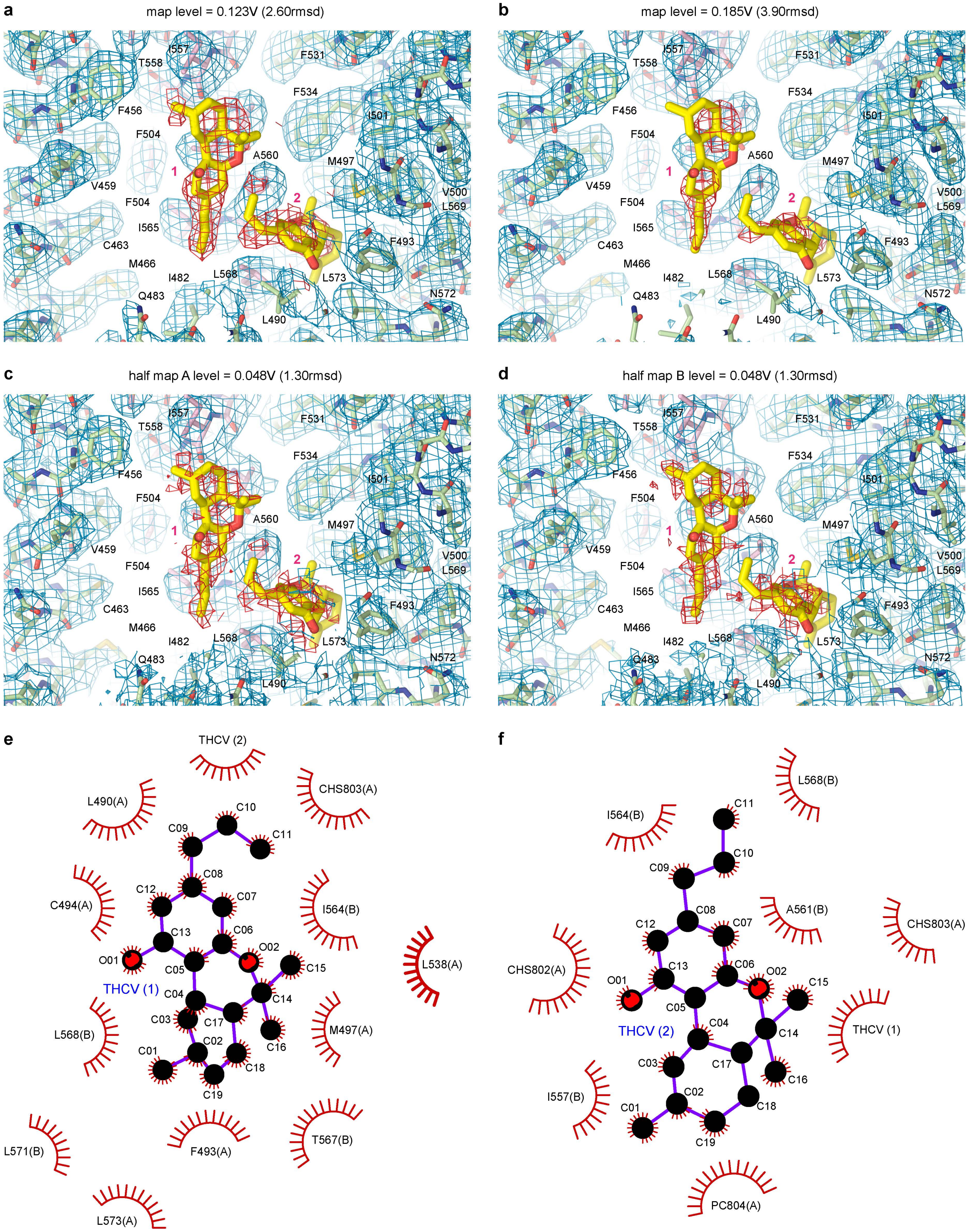

Supplementary Fig. 3 | Maps and LigPlot analysis for THCV binding. a–d Maps for THCV (red mesh) and surrounding protein (blue mesh). Shown are the final map at different contour levels (**a**,**b**) as well as half maps A (**c**) and B (**d**). **e**,**f** LigPlot analysis for THCV sites 1 (**e**) and 2 (**f**).

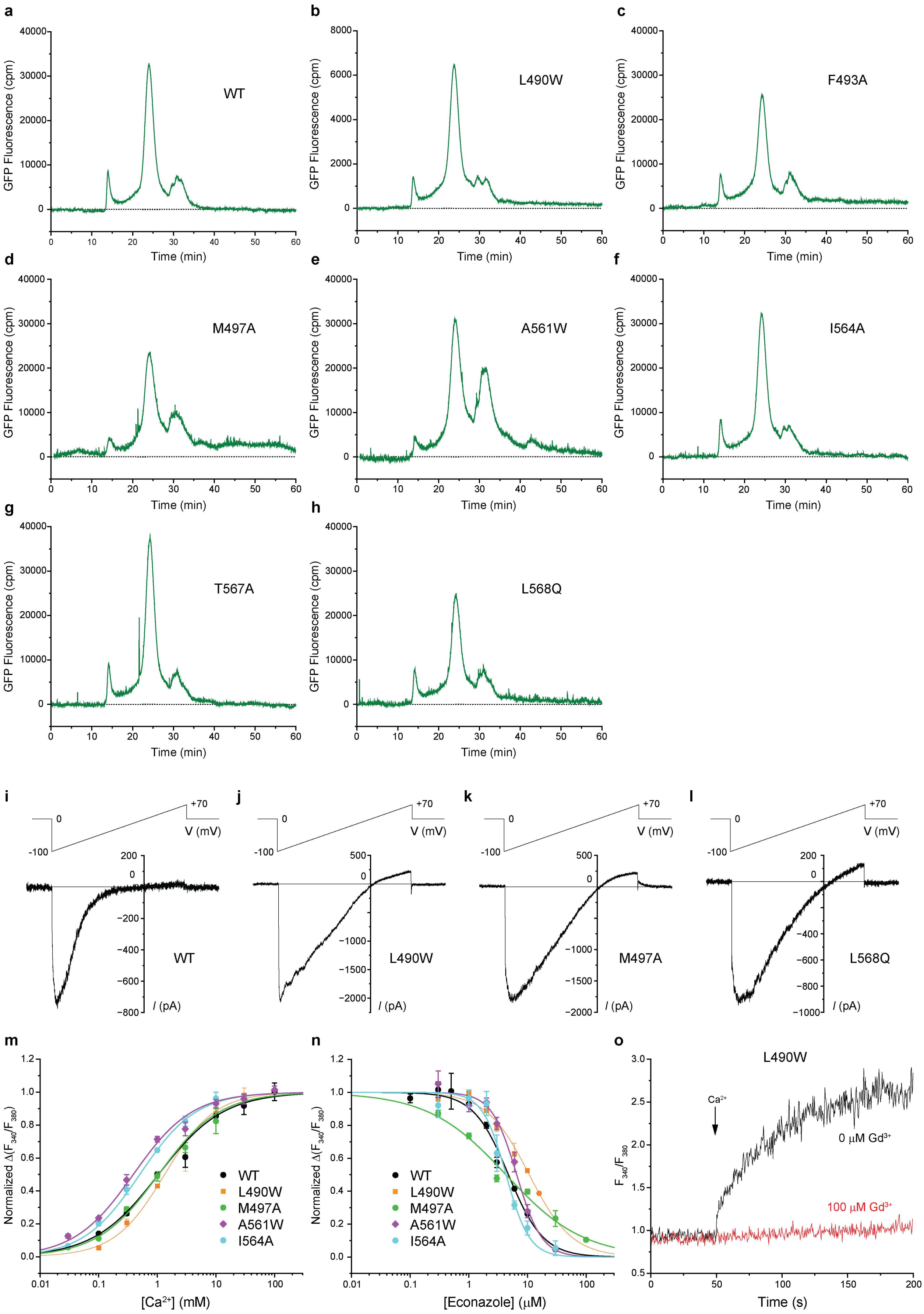

a
WT
b
L490W
c
F493A
d
M497A
e
A561W
f
I564A
g
T567A
h
L568Q
GFP Fluorescence (cpm)
Time (min)
i
WT
j
L490W
k
M497A
l
L568Q
V (mV)
I (pA)
+70
0
-100
m
Normalized Δ(F340/F380)
[Ca2+] (mM)
WT
L490W
M497A
A561W
I564A
n
Normalized Δ(F340/F380)
[Econazole] (μM)
WT
L490W
M497A
A561W
I564A
o
L490W
Ca2+
0 μM Gd3+
100 μM Gd3+
F340/F380
Time (s)

Supplementary Fig. 4 | FSEC and functional characterization of hTRPV6 mutants. a–h FSEC profiles for wild type (**a**) and mutant (**b-h**) hTRPV6. Note the major tetrameric peak eluting at ~25 min. **i–l** Whole-cell patch-clamp currents recorded from HEK 293 cells expressing wild type (**i**) or mutant (**j–l**) hTRPV6 in response to −100 to 70 mV voltage ramp. Examples are representatives of currents recorded from 3 to 20 different cells in two to four independent transfections. **m** Concentration-dependencies of Ca^{2+} uptake activation by calcium fitted with the logistic equation (curves through the points), with the EC_{50} values of 1.05 ± 0.03 mM (n = 3) for wild type hTRPV6, 1.33 ± 0.04 mM (n = 3) for L490W, 1.05 ± 0.12 mM (n = 3) for M497A, 0.39 ± 0.02 mM (n = 3) for A561W, and 0.50 ± 0.02 mM (n = 3) for I564A. Data points are mean ± SEM. Source data are provided as a Source Data file. **n** Concentration-dependencies of 10 mM Ca^{2+}-induced Ca^{2+} uptake inhibition by econazole fitted with the logistic equation (curves through the points), with the IC_{50} values of 4.73 ± 0.13 μM (n = 9) for wild type hTRPV6, 10.7 ± 0.7 μM (n = 3) for L490W, 4.29 ± 0.58 μM (n = 3) for M497A, 6.55 ± 0.34 μM (n = 3) for A561W, and 4.26 ± 0.26 μM (n = 3) for I564A. Data points are mean ± SEM. Source data are provided as a Source Data file. **o** Representative ratiometric Fura-2-based fluorescence measurements of changes in intracellular Ca^{2+} for HEK 293S GnTI^{-} cells expressing L490W mutant hTRPV6 channels. The changes in the fluorescence intensity ratio at 340 and 380 nm (F_{340}/F_{380}) were monitored in response to application of 10 mM Ca^{2+} (arrow) after pre-incubation of cells without and with 100 μM of ion channel blocker Gd^{3+}. The experiment was repeated independently three times with similar results.

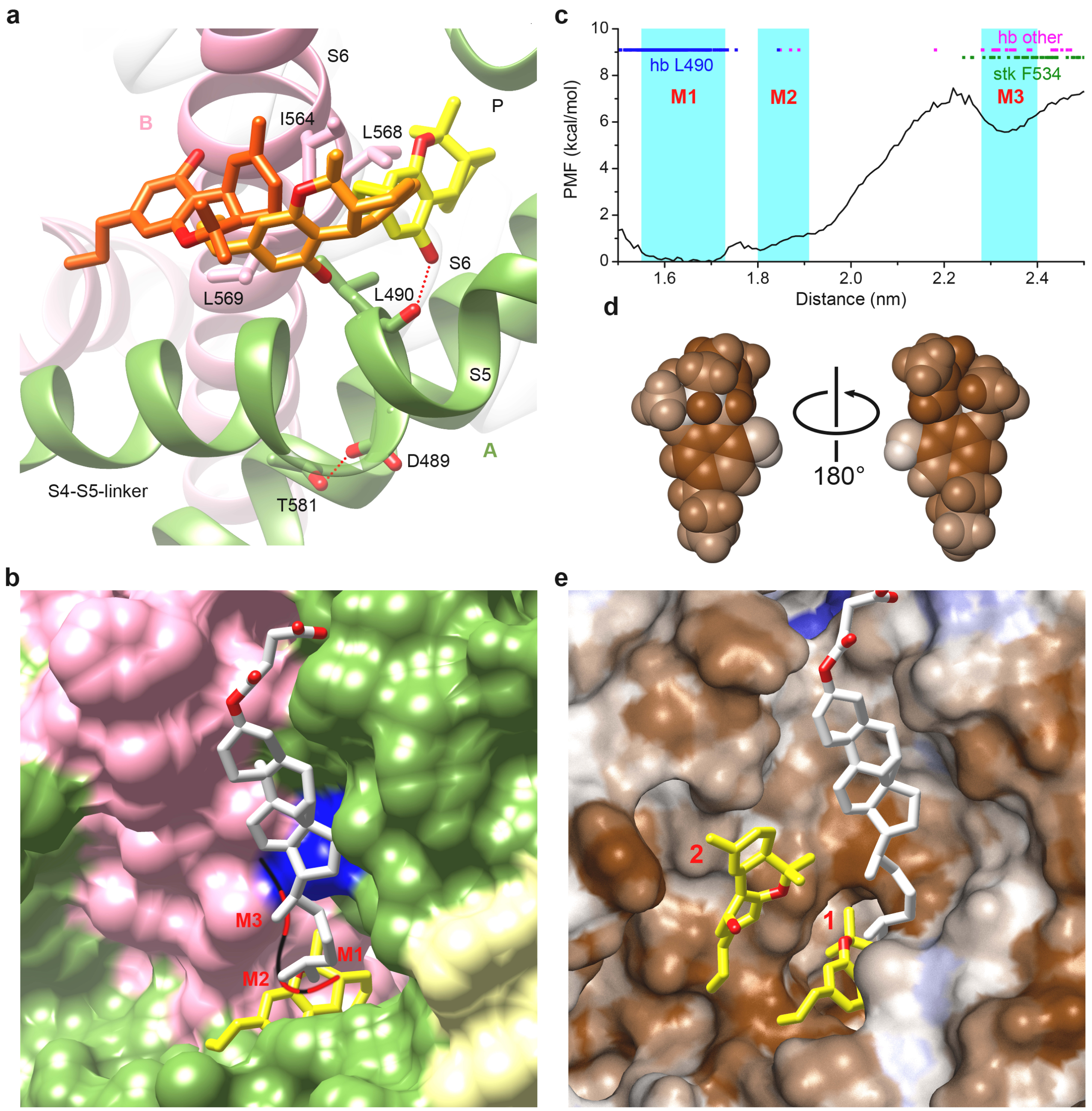

Supplementary Fig. 5 | Additional details of MD simulation of THCV binding to TRPV6. a THCV leaving site1 of the open TRPV6 along the horizontal pathway. Yellow, orange, and red sticks show three successive MD-snapshots of THCV along the leaving path. Cryo-EM model of protein is shown as green (subunit A) and pink (subunit B) cartoon. Hydrogen bonds between THCV-L490 and T581-D489 are shown as red dotted lines. **b** THCV vertical pathway is shown as a black curve, with energy minima along the pathway indicated by red segments M1, M2 and M3. Surface of subunit A, subunit B and F534 is colored green, pink and blue, respectively. Molecules of CHS (white) and THCV (yellow) at site 1 are shown as sticks. **c** Potential of mean force calculated along the vertical pathway, with the energy minima M1, M2, and M3 highlighted in pink. Blue, pink, and green lines indicate hydrogen bonds between THCV and L490, between THCV and other residues, and π-stacking interaction between THCV and F534, respectively. **d** THCV molecule with atoms colored according to the molecular hydrophobicity potential (MHP) on their surface, where hydrophobic and hydrophilic surfaces are shown in brown and blue, respectively. **e** Closeup view of THCV binding pocket, with protein surface colored according to the MHP. Molecules of THCV (yellow) and CHS (white) are shown in sticks.

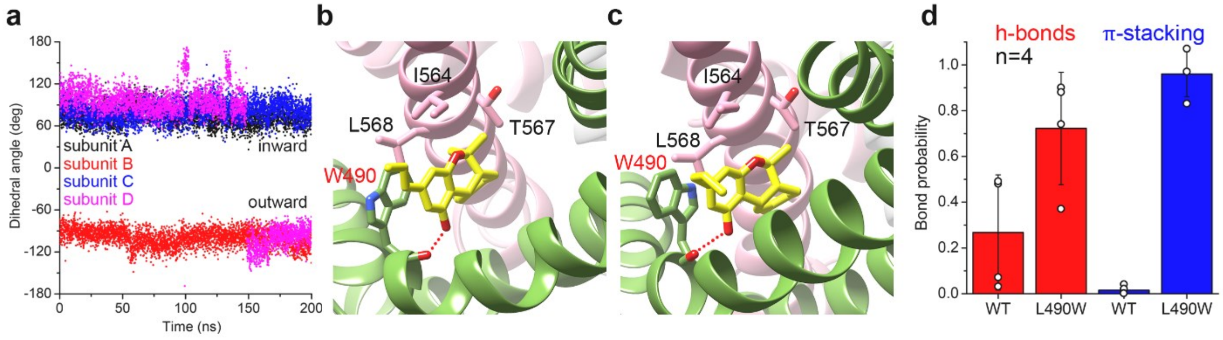

Supplementary Fig. 6 | MD simulation of THCV binding to the TRPV6-L490W mutant. a Orientations of side chains of residues W490 during MD simulations. Dihedral angles for residues in four TRPV6 subunits are shown with different colors. The inward and outward orientations of W490 correspond to the dihedral angle (C_{α}-C_{β}-C_{γ}-$C_{\delta 2}$) values of 80° and –110°, respectively. **b,c** Close-up views of site 1 in the L490W mutant, with the tryptophan side chain in inward (**b**) and outward (**c**) orientations. **d** Average probability of hydrogen bond formation between THCV and residue 490 (red) and average probability of π-stacking interaction between THCV and aromatic residues in MD simulations of the wild-type and mutant channels. Data in **d** are mean over four independent THCV molecules, errors are ± SD.

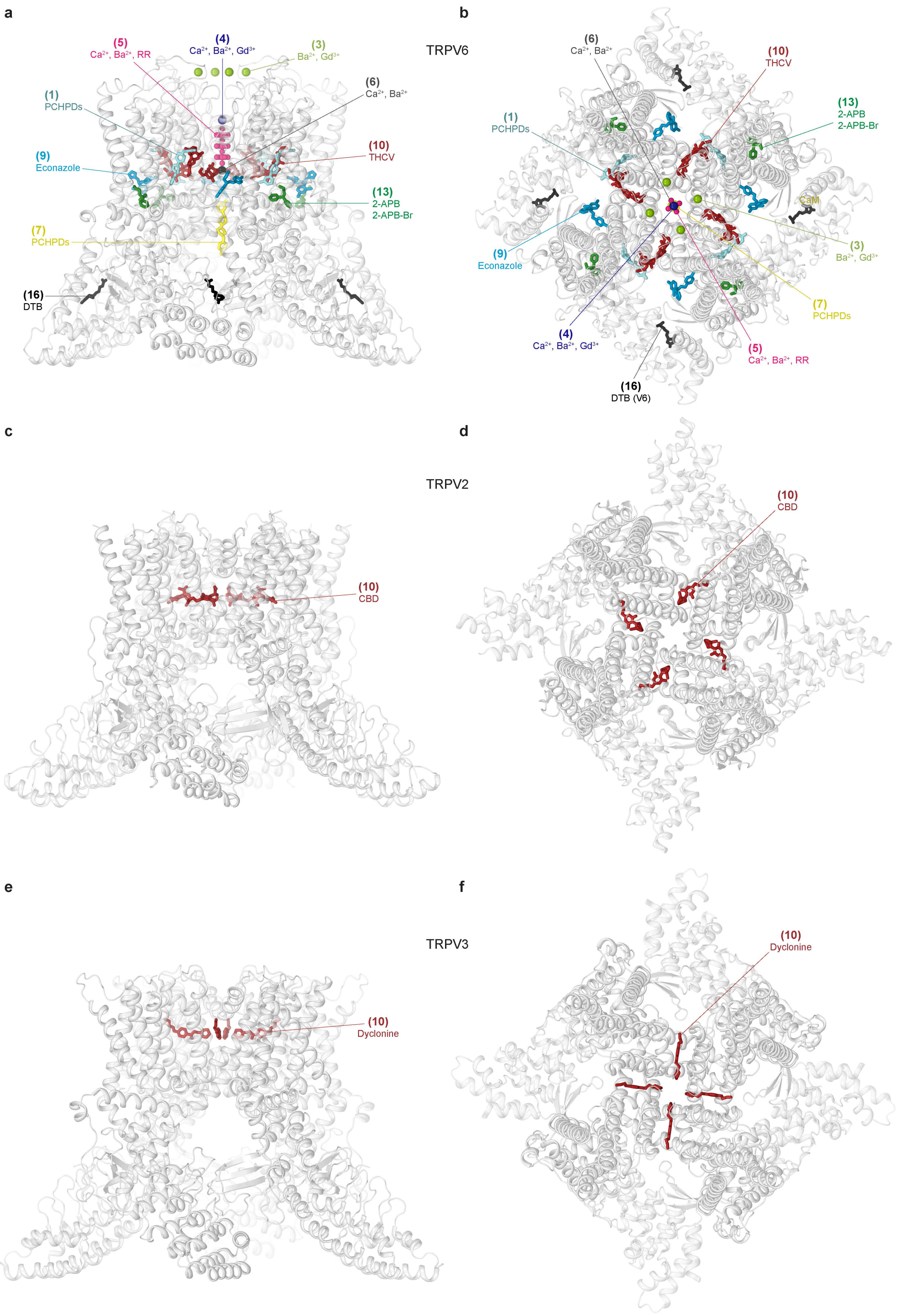

Supplementary Fig. 7 | THCV versus other TRPV6 ligands and portal site ligands in vanilloid-subfamily TRP channels. a,b Side (**a**) and top (**b**) views of hTRPV6$_{THCV}$ (grey cartoon) with different TRPV6 binding sites mapped by differently colored ligands and numbered as described[1]. The ligands include Ca^{2+}, Ba^{2+} and Gd^{3+} ions[2,3], desthiobiotin (DTB)[2], 2-aminoethoxydiphenyl borate (2-APB) and its brominated analog 2-APB-Br[4], (4-phenylcyclohexyl)piperazine derivatives (PCHPDs)[5], ruthenium red (RR)[6], and econazole[6]. **c-d** Side (**c**) and top (**d**) views of rat TRPV2 (PDB ID: 6U88)[7] with bound cannabidiol (CBD) molecule at the portal site shown in brown. **e,f** Side (**e**) and top (**f**) views of mouse TRPV3 (PDB ID: 7UGG) with bound dyclonine molecule at the portal site shown in brown.

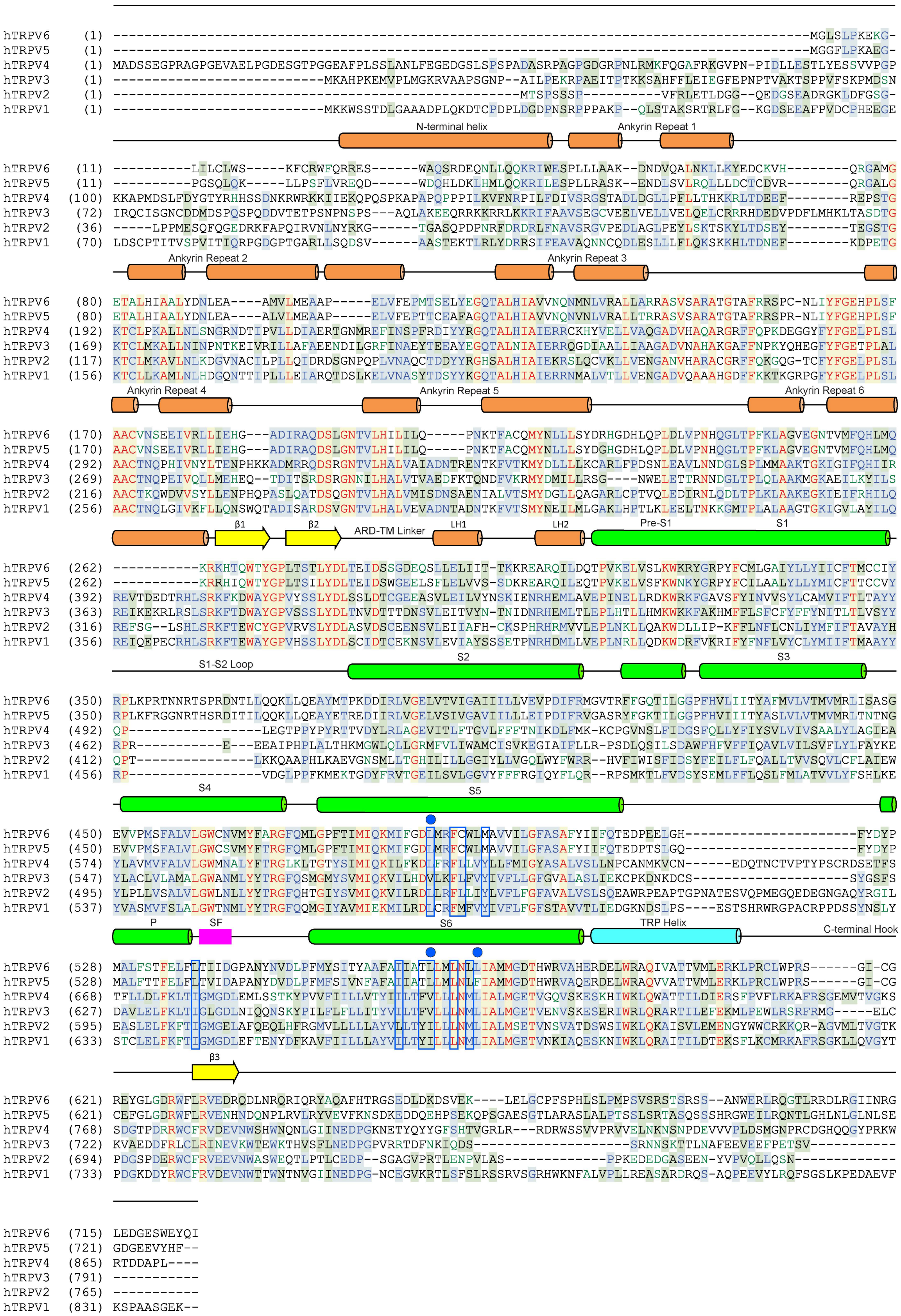

Supplementary Fig. 8 | Sequence alignment of human vanilloid-subfamily TRP channels. Secondary structure elements are depicted above the sequence as cylinders (α-helices), arrows (β-strands), and lines (loops). The pink rectangle marks the selectivity filter. Blue boxes indicate residues involved in THCV binding to site 1. Mutated residues are labeled with blue circles.

Supplementary Table 1 | Cryo-EM data collection, refinement and validation statistics

**Structure**	hTRPV6$_{THCV}$
EMDB accession code	EMD-40676
PDB accession code	8SP8
**Data collection and processing**	
Magnification	130,000x
Voltage (kV)	300
Electron exposure (e^{-}Å^{-2})	60
Defocus range (μm)	−0.8 to −2.0
Reported pixel size (Å)	0.825
Exposures (no.)	6,699
**Processing software**	
Motion correction	cryoSPARC v3.3
CTF estimation	cryoSPARC v3.3
Platform software for particle picking	cryoSPARC v3.3
Software for 2D/3D class. & Refinements	cryoSPARC v3.3
Symmetry imposed	C4
Initial particle images (no.)	4,502,785
Final particle images (no.)	213,249
Map resolution (Å)	2.79
FSC 0.143	
**Refinement**	
Initial models used (PDB code)	7S8C
Model resolution (Å)	2.79
FSC threshold	0.143
Map sharpening *B* factor (Å^2)	−111.0
**Model composition**	
Non-hydrogen atoms	21,082
Protein residues	2,456
Ligands	42
B factors (Å^2)	
Protein	92.79
Ligands	33.66
R.m.s. deviations	
Bond lengths (Å)	0.010
Bond angles (°)	1.066
**Validation**	
MolProbity score	1.55
Clash score, all atoms	1.93
Poor rotamers (%)	0.00
Ramachandran plot	
Favored (%)	91.80
Allowed (%)	8.20
Disallowed (%)	0.00

Supplementary Table 2 | List of MD runs

Run name	Simulation duration	Ligand starting position	Description
fwd_run1	40+200 ns*	pose 1 (like in cryo-EM model)	Forward orientation of THCV molecules in sites 1 of $TRPV6_{THCV}$.
fwd_run2	40+50 ns*	pose 2	
fwd_run3	40+50 ns*	pose 3	
fwd_run4	40+50 ns*	pose 4	
bwd_run1	40+50 ns*	pose 5	Backward orientation of THCV molecules in sites 1 of $TRPV6_{THCV}$.
bwd_run2	40+50 ns*	pose 6	
bwd_run3	40+50 ns*	pose 7	
bwd_run4	40+50 ns*	pose 8	
open_run	40+50 ns*	pose 1 (like in cryo-EM model)	Forward orientation of THCV molecules in sites 1 of open TRPV6 (PDB ID 7S88).
L490W_run	40+200 ns*	pose 1 (like in cryo-EM model)	Forward orientation of THCV molecules in sites 1 of L490W mutant of $TRPV6_{THCV}$.
pulling_run	50 ns	pose 1 (like in cryo-EM model)	Pulling of THCVs from sites 1 of $TRPV6_{THCV}$.
umb_runs	32 × 5 ns	poses from pulling_run	32 starting configurations for umbrella sampling windows, two THCV molecules were chosen for the umbrella simulation: lig2 (vertical pathway) and lig4 (horizontal pathway).

* 40 ns of equilibration were included in the analyzed data set.

Supplementary References

1 Yelshanskaya, M. V. & Sobolevsky, A. I. Ligand-Binding Sites in Vanilloid-Subtype TRP Channels. *Front Pharmacol* **13**, 900623, doi:10.3389/fphar.2022.900623 (2022).

2 Saotome, K., Singh, A. K., Yelshanskaya, M. V. & Sobolevsky, A. I. Crystal structure of the epithelial calcium channel TRPV6. *Nature* **534**, 506-511, doi:10.1038/nature17975 nature17975 [pii] (2016).

3 Singh, A. K., Saotome, K. & Sobolevsky, A. I. Swapping of transmembrane domains in the epithelial calcium channel TRPV6. *Sci Rep* **7**, 10669, doi:10.1038/s41598-017-10993-9 (2017).

4 Singh, A. K., Saotome, K., McGoldrick, L. L. & Sobolevsky, A. I. Structural bases of TRP channel TRPV6 allosteric modulation by 2-APB. *Nat Commun* **9**, 2465, doi:10.1038/s41467-018-04828-y (2018).

5 Bhardwaj, R. *et al.* Inactivation-mimicking block of the epithelial calcium channel TRPV6. *Sci Adv* **6**, eabe1508, doi:10.1126/sciadv.abe1508 (2020).

6 Neuberger, A., Nadezhdin, K. D. & Sobolevsky, A. I. Structural mechanisms of TRPV6 inhibition by ruthenium red and econazole. *Nat Commun* **12**, 6284, doi:10.1038/s41467-021-26608-x (2021).

7 Pumroy, R. A. *et al.* Molecular mechanism of TRPV2 channel modulation by cannabidiol. *Elife* **8**, e48792, doi:10.7554/eLife.48792 (2019).
