# Supplementary material for: Molecular pathway and structural mechanism of human oncochannel TRPV6 inhibition by the phytocannabinoid tetrahydrocannabivarin
Source: Nat Commun. 2023 Aug 2;14:4630. doi: 10.1038/s41467-023-40362-2 (PMC10397291; doi:10.1038/s41467-023-40362-2)
Supplement: Supplementary file 3 — Description of Additional Supplementary Files [file 41467_2023_40362_MOESM3_ESM.pdf]

Description of Additional Supplementary Files

File name: Supplementary Data 1

Description: Coordinates of the protein obtained in MD simulations

File name: Supplementary Data 2

Description: Coordinates of the ligands obtained in MD simulations
