# Supplementary material for: Molecular pathway and structural mechanism of human oncochannel TRPV6 inhibition by the phytocannabinoid tetrahydrocannabivarin
Source: Nat Commun. 2023 Aug 2;14:4630. doi: 10.1038/s41467-023-40362-2 (PMC10397291; doi:10.1038/s41467-023-40362-2)
Supplement: Supplementary file 5 — Supplementary Data 2 [file 41467_2023_40362_MOESM5_ESM.zip › Supplementary Data 2/List of MD runs.docx]

List of MD runs

| **Run name** | **Simulation duration** | **Ligand starting position** | **Description** |
| --- | --- | --- | --- |
| fwd_run1 | 40+200 ns* | pose 1 (like in cryo-EM model) | Forward orientation of THCV molecules in sites 1 of TRPV6_THCV_. |
| fwd_run2 | 40+50 ns* | pose 2 |  |
| fwd_run3 | 40+50 ns* | pose 3 |  |
| fwd_run4 | 40+50 ns* | pose 4 |  |
| bwd_run1 | 40+50 ns* | pose 5 | Backward orientation of THCV molecules in sites 1 of TRPV6_THCV_. |
| bwd_run2 | 40+50 ns* | pose 6 |  |
| bwd_run3 | 40+50 ns* | pose 7 |  |
| bwd_run4 | 40+50 ns* | pose 8 |  |
| open_run | 40+50 ns* | pose 1 (like in cryo-EM model) | Forward orientation of THCV molecules in sites 1 of open TRPV6 (PDB ID 7S88). |
| L490W_run | 40+200 ns* | pose 1 (like in cryo-EM model) | Forward orientation of THCV molecules in sites 1 of L490W mutant of TRPV6_THCV_. |
| pulling_run | 50 ns | pose 1 (like in cryo-EM model) | Pulling of THCVs from sites 1 of TRPV6_THCV_. |
| umb_runs | 32 × 5 ns | poses from pulling_run | 32 starting configurations for umbrella sampling windows, two THCV molecules were chosen for the umbrella simulation: lig2 (vertical pathway) and lig4 (horizontal pathway). |

* – 40 ns of equilibration were included to analyzed data set.
