# Supplementary material for: Molecular pathway and structural mechanism of human oncochannel TRPV6 inhibition by the phytocannabinoid tetrahydrocannabivarin
Source: Nat Commun. 2023 Aug 2;14:4630. doi: 10.1038/s41467-023-40362-2 (PMC10397291; doi:10.1038/s41467-023-40362-2)
Supplement: Supplementary file 6 — Reporting Summary [file 41467_2023_40362_MOESM6_ESM.pdf]

nature portfolio

Corresponding author(s): Alexander I. Sobolevsky

Last updated by author(s): Jul 5, 2023

Reporting Summary

Nature Portfolio wishes to improve the reproducibility of the work that we publish. This form provides structure for consistency and transparency in reporting. For further information on Nature Portfolio policies, see our Editorial Policies and the Editorial Policy Checklist.

Statistics

For all statistical analyses, confirm that the following items are present in the figure legend, table legend, main text, or Methods section.

n/a	Confirmed	
☐	☒	The exact sample size (n) for each experimental group/condition, given as a discrete number and unit of measurement
☐	☒	A statement on whether measurements were taken from distinct samples or whether the same sample was measured repeatedly
☐	☒	The statistical test(s) used AND whether they are one- or two-sided<br>*Only common tests should be described solely by name; describe more complex techniques in the Methods section.*
☒	☐	A description of all covariates tested
☒	☐	A description of any assumptions or corrections, such as tests of normality and adjustment for multiple comparisons
☐	☒	A full description of the statistical parameters including central tendency (e.g. means) or other basic estimates (e.g. regression coefficient) AND variation (e.g. standard deviation) or associated estimates of uncertainty (e.g. confidence intervals)
☐	☒	For null hypothesis testing, the test statistic (e.g. F, t, r) with confidence intervals, effect sizes, degrees of freedom and P value noted<br>*Give P values as exact values whenever suitable.*
☒	☐	For Bayesian analysis, information on the choice of priors and Markov chain Monte Carlo settings
☒	☐	For hierarchical and complex designs, identification of the appropriate level for tests and full reporting of outcomes
☒	☐	Estimates of effect sizes (e.g. Cohen's d, Pearson's r), indicating how they were calculated

Our web collection on statistics for biologists contains articles on many of the points above.

Software and code

Policy information about availability of computer code

Data collection	SerialEM 4.0, pCLAMP 11
Data analysis	cryoSPARC 3.3, UCSF Chimera 1.17, UCSF ChimeraX 1.5, COOT 0.9.8.1, PHENIX 1.19.2, PyMOL 2.5.2, Origin 9.1.0, HOLE 2.1, GAUSSIAN09, Revision A.01, GROMACS 2021.4 package, Charmm36 force field (Charmm 2020), TIP3P water model 8.4.3, LINCS algorithm (2023)

For manuscripts utilizing custom algorithms or software that are central to the research but not yet described in published literature, software must be made available to editors and reviewers. We strongly encourage code deposition in a community repository (e.g. GitHub). See the Nature Portfolio guidelines for submitting code & software for further information.

Data

Policy information about availability of data

All manuscripts must include a data availability statement. This statement should provide the following information, where applicable:

- Accession codes, unique identifiers, or web links for publicly available datasets
- A description of any restrictions on data availability
- For clinical datasets or third party data, please ensure that the statement adheres to our policy

All data needed to evaluate the conclusions of the paper are present in the paper or the Supplementary Data. The cryo-EM density map of hTRPV6 in complex with THCV was deposited to the Electron Microscopy Data Bank (EMDB) under the accession codes EMD-40676 (https://www.emdataresource.org/EMD-40676). The atomic coordinates have been deposited to the Protein Data Bank (PDB) under the accession codes 8SP8 (https://www.rcsb.org/structure/8SP8, see Supplementary

Table 1). THCV coordinate (THCV_conf2.mol2) and topology (THCV.itp and extra.itp) files used in MD simulations are available as supplementary files.
The accession codes for previously published structures that were used for model building, MD simulations, and/or illustrations: 7S88 (https://doi.org/10.2210/pdb7S88/pdb), 7S89 (https://doi.org/10.2210/pdb7S89/pdb), 7S8B (https://doi.org/10.2210/pdb7S8B/pdb), 7S8C (https://doi.org/10.2210/pdb7S8C/pdb), 6U88 (https://doi.org/10.2210/pdb6U88/pdb) and 7UGG (https://doi.org/10.2210/pdb7UGG/pdb).
PDB THCV coordinate (THCV_conf2.mol2) and topology (THCV.itp and extra.itp) files used in MD simulations and coordinates of the protein and ligands obtained in MD simulations (see also Supplementary Table 2) are available as supplementary data files. The statistics of THCV-protein interactions obtained in MD simulations is available in the source data file. All other data are available from the corresponding author upon request. Source data are provided with this paper.

Research involving human participants, their data, or biological material

Policy information about studies with human participants or human data. See also policy information about sex, gender (identity/presentation), and sexual orientation and race, ethnicity and racism.

Reporting on sex and gender	not applicable
Reporting on race, ethnicity, or other socially relevant groupings	not applicable
Population characteristics	not applicable
Recruitment	not applicable
Ethics oversight	not applicable

Note that full information on the approval of the study protocol must also be provided in the manuscript.

Field-specific reporting

Please select the one below that is the best fit for your research. If you are not sure, read the appropriate sections before making your selection.

☒ Life sciences ☐ Behavioural & social sciences ☐ Ecological, evolutionary & environmental sciences

For a reference copy of the document with all sections, see nature.com/documents/nr-reporting-summary-flat.pdf

Life sciences study design

All studies must disclose on these points even when the disclosure is negative.

Sample size	The amount of cryo-EM data collected in this study was limited by time allocation at the microscope. Based on our previous collections with this protein (e.g., Neuberger et al., 2021 [https://doi.org/10.1038/s41467-021-26608-x] and Neuberger et al., 2023 [https://doi.org/10.1038/s41467-023-38352-5]), a collection of 3,000 exposure and above was expected to yield a high-resolution reconstruction of the channel protein as well as to resolve the ligand. 3,516 exposures were collected in a one-day session and it was deemed unnecessary to collect for an additional day. Amount of MD data collected was limited by time allocation and computational resources available (see MD data collected in Neuberger et al., 2023 [https://doi.org/10.1038/s41467-023-38352-5]). Single recordings were performed in FSEC experiments due to the high reproducibility of the data using this method (see Neuberger et al., 2021 [https://doi.org/10.1016/bs.mie.2021.02.006]). For Fura-2 AM and patch-clamp experiments, we performed all measurements three times or more, but even triplicate measurements were sufficient given the high reproducibility of results (see also Neuberger et al., 2023 [https://doi.org/10.1038/s41467-023-38352-5]).
Data exclusions	No data has been excluded.
Replication	No replication attempts have failed. The cryo-EM data collection was consistent from the beginning to the end. A replication of the cryo-EM data collection was therefore not necessary or economically viable/justifiable. In Fura-2 AM and patch-clamp experiments, we made at least three independent replicates for each construct. No replication was performed in FSEC experiments due to the high reproducibility of the data using this method.
Randomization	Samples were not randomized; it is not technically or practically feasible to do so for cryo-EM, Fura-2 AM, patch-clamp or the conducted molecular dynamic simulation studies. Covariant control is not economically viable/justifiable in cryo-EM data collections or MD simulations. Covariant control was also not possible for Fura-2 AM, patch-clamp or FSEC studies due to the need to transfect cells with predetermined viruses and optimize protein expression for individual constructs.
Blinding	Researchers were not blinded; it is not technically or practically feasible to do so for cryo-EM, Fura-2 AM, patch-clamp or the conducted molecular dynamic simulation studies. It is not economically viable/justifiable to blind cryo-EM collections or MD simulations. For Fura-2 AM, patch-clamp or FSEC studies, researchers conducting the studies were also in charge of cell as well as protein expression optimization for individual constructs in order to achieve recordings on transfected cells in these studies. These circumstances made blinding not possible.

Reporting for specific materials, systems and methods

We require information from authors about some types of materials, experimental systems and methods used in many studies. Here, indicate whether each material, system or method listed is relevant to your study. If you are not sure if a list item applies to your research, read the appropriate section before selecting a response.

Materials & experimental systems

n/a	Involved in the study
☒	☐ Antibodies
☐	☒ Eukaryotic cell lines
☒	☐ Palaeontology and archaeology
☒	☐ Animals and other organisms
☒	☐ Clinical data
☒	☐ Dual use research of concern
☒	☐ Plants

Methods

n/a	Involved in the study
☒	☐ ChIP-seq
☒	☐ Flow cytometry
☒	☐ MRI-based neuroimaging

Eukaryotic cell lines

Policy information about cell lines and Sex and Gender in Research

Cell line source(s)	HEK293 S GnTI-, ATCC #CRL-3022<br>HEK293, ATCC #CRL-1573<br>Sf9, Gibco, Cat#12659017
Authentication	None of the cell lines used (including HEK 293 S GnTI-, HEK293 and Sf9 cells) have been authenticated.
Mycoplasma contamination	The cell lines used (including HEK 293 S GnTI-, HEK293 and Sf9 cells) have been tested for mycoplasma contamination by the providers (negative results) but have not been retested in the lab.
Commonly misidentified lines<br>(See ICLAC register)	No commonly misidentified lines were used in this study.
